# Midlife predictors of active and healthy aging (AHA) among older businessmen

**DOI:** 10.1007/s40520-018-1100-0

**Published:** 2018-12-24

**Authors:** Annele Urtamo, Emmi Huohvanainen, Kaisu H. Pitkälä, Timo E. Strandberg

**Affiliations:** 1 Department of General Practice and Primary Health Care, and Helsinki University Central Hospital, Unit of Primary Health Care, University of Helsinki, Helsinki, Finland; 2 Clinicum, and Helsinki University Hospital, University of Helsinki, Helsinki, Finland; 3 Center for Life Course Health Research, University of Oulu, Oulu, Finland

**Keywords:** Successful aging, Longevity, Life course, Self-rated health, Cardiovascular risk factors

## Abstract

**Background:**

Active and healthy aging (AHA) is an important phenomenon in aging societies.

**Aims:**

Our aim was to investigate midlife predictors of AHA in a socioeconomically homogenous male cohort.

**Methods:**

In 2010, AHA was defined in the Helsinki Businessmen Study (men born in 1919–1934) with six criteria: (1) being alive, (2) responding to the mailed survey, (3) no reported cognitive problems, (4) feeling of happiness, (5) no difficulties in activities of daily living (ADL), and (6) no significant chronic diseases. Midlife factors were assessed in 1974 (*n* = 1759, mean age 47 years). Of the survivors in 2010 (*n* = 839), 10.0% (*n* = 84) fulfilled all AHA criteria, whilst 13.7% (*n* = 115) had chronic diseases but fulfilled other five criteria. Midlife predictors of AHA were analyzed with logistic models.

**Results:**

Of the midlife factors, smoking [Odds ratio (OR) 0.44, 95% confidence interval (CI) 0.25–0.77], higher body mass index (BMI) (OR 0.75, 0.59–0.96), and higher total cholesterol (OR 0.76, 0.60–0.97) prevented significantly full AHA criteria, whereas higher self-rated health (SRH) (OR 1.73, 1.07–2.80) predicted significantly of fulfilling all AHA criteria. Midlife smoking (OR 0.87, 0.84–0.91), higher BMI (OR 0.73, 0.61–0.86), and higher alcohol consumption (OR 0.73, 0.60–0.90) prevented significantly of fulfilling the five AHA criteria with chronic diseases, and higher SRH (OR 1.90, 1.37–2.63) predicted significantly the five AHA criteria (chronic diseases present).

**Discussion:**

Our study suggests that midlife factors, especially good SRH and low levels of cardiovascular risk factors, are associated with AHA.

**Conclusions:**

The study emphasizes the importance of life-course predictors of healthy aging.

## Introduction

Maintaining a good health and function in old age is an important target in aging societies. Rowe and Kahn [1, 2] have defined successful aging as an optimal psychological, physical and social functioning in old age without major diseases. Young et al. [3] have proposed an alternative model, which captures the possibility to compensate physiological limitations by psychological and social dimensions. The main consideration has been how to minimize the years of morbidity and disabilities in old age, and, thus, expand healthy and functional life span [4, 5]. Furthermore, various definitions and assessments of successful and healthy aging have been used in studies [6]. In a previous analysis of Helsinki Businessmen Study (HBS) cohort, active and healthy aging (AHA) was defined as an ability to perform daily activities and feeling happy without cognitive or functional impairments, and major chronic diseases [7].

Previous studies with varying age profiles indicate that lifestyle and biological risk factors in midlife are associated with longevity and various aspects of healthy life in old age [8–15]. In the HBS cohort, we have observed the relationship between cardiovascular risk factors in midlife and health-related quality of life (HRQoL) in old age [16–19]. In addition, studies have shown an association between midlife value priorities [20], behavioral factors [15], and self-rated health (SRH) [21] with late-life frailty and mortality. In a cross-sectional study of the HBS cohort, < 5% met the criteria for AHA, which was associated with physical functioning and subjective health [7].

To our knowledge, there are no studies exploring midlife predictors of AHA, which have extremely long follow-up, and also take mortality before old age into account. In addition, less is known about predictors of AHA in the presence of chronic diseases. We investigated these in the HBS cohort.

## Design and methods

### Study population

**Fig. 1:**
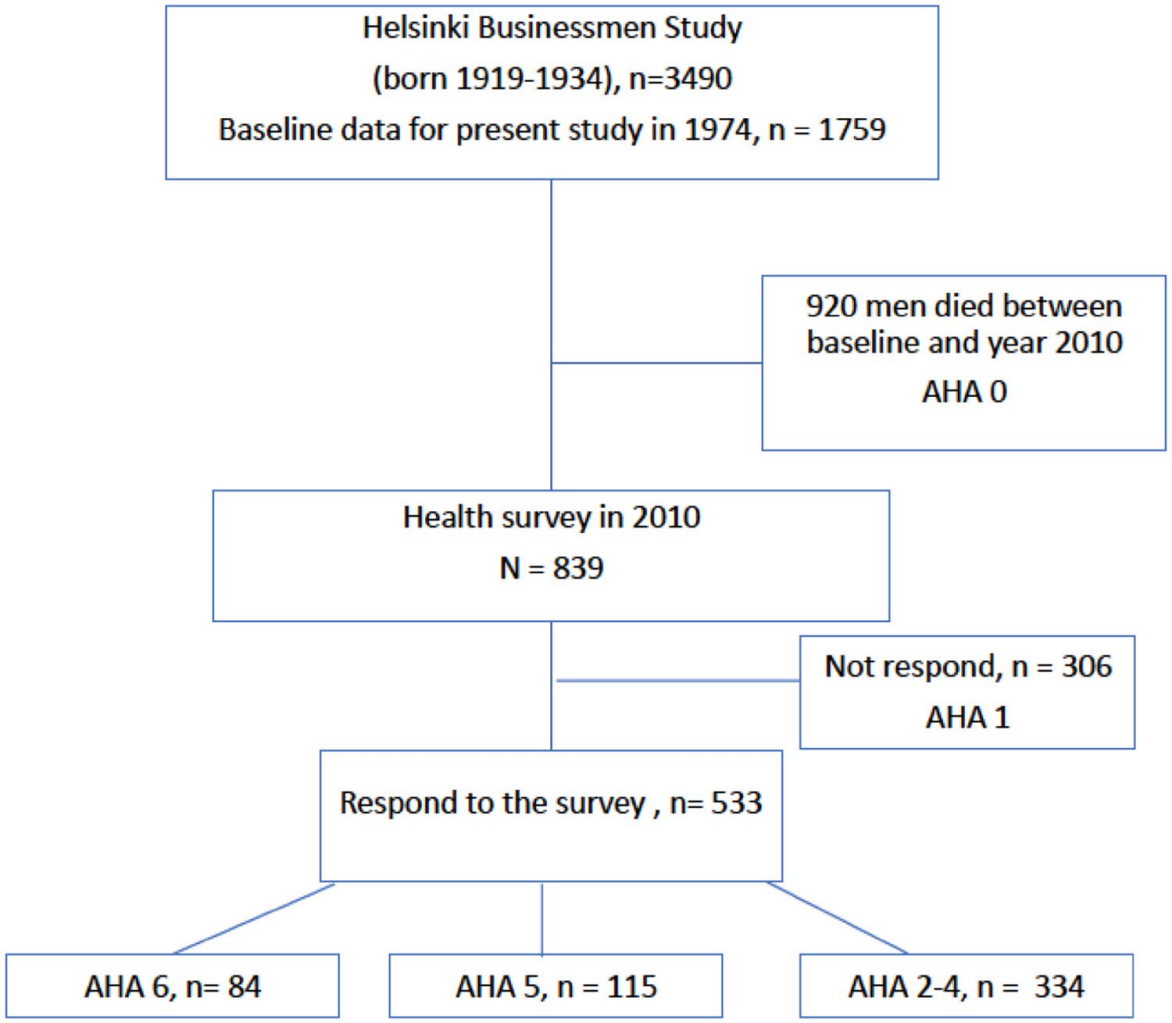
Flow chart of the study

HBS is a longitudinal study of initially healthy Finnish businessmen and executives born between 1919 and 1934 (original *n* = 3490). Details of the study cohort have been described earlier [22]. The cohort has been followed-up for over 50 years with mailed questionnaires, clinical and laboratory assessments. For the present study, the relevant baseline data in the year 1974 were available from those men who were certified to be clinically healthy and without chronic medications in their midlife (*n* = 1759 men, 50.4% of original cohort). The follow-up was approved by the Ethics Committee of the Helsinki University Hospital, Department of Medicine, and all participants had provided relevant informed consents. The flow chart of study is presented in Fig. 1.

### Baseline measurements

In 1974, the men (*n* = 1759, mean age 47 years) were assessed with clinical and laboratory examinations including body mass index (BMI, kg/m^2^), systolic and diastolic blood pressure (mmHg), total serum cholesterol (mmol/L) and triglycerides (mmol/L). In addition, the status of smoking and consumption of coffee (cups/day) and alcohol (portions/week) were asked. Self-rated health (SRH) was assessed with similar wording as used in the Whitehall II study [23]. SRH and self-rated fitness (SRF) were assessed by asking “What do you think about your present state of health/fitness”; is it “very good”, “fairly good”, “average”, “fairly poor” or “very poor”. The statuses such as “very good” and “fairly good” were combined in the analyses of SRH and SRF.

### Definition of AHA in 2010

In 2010, 839 men of the present study responded to mailed survey, and the criteria of AHA were defined by Rantanen et al. [7] as: (1) being alive, (2) responding to the mailed questionnaire survey (reflects social functioning), (3) no subjective cognitive problems (self-reported in the survey according to the memory item in the Clinical Dementia Rating; reflects cognitive functioning), (4) feeling of happiness (self-reported in the survey, RAND-36 item; reflects psychological functioning), (5) no difficulties in activities of daily living (ADL) (self-reported in the survey; reflects physical functioning), and (6) no significant chronic diseases (cardiovascular diseases, Parkinson disease, Alzheimer disease, rheumatoid arthritis, cancer, obstructive pulmonary disease, diabetes; self-reported in the survey; reflects physical health).

For analyses, AHA was defined as follows: (AHA 0) deceased; (AHA 1) alive, but not responding to the mailed survey; (AHA 2–4) responding to the mailed survey plus one to two of the following criteria: no cognitive problems, feeling of happiness, no ADL difficulties; (AHA 5) as all criteria above plus possible chronic diseases, (AHA 6) as above but without chronic diseases.

### Mortality

The dates of death were drawn from the national Population Information System. Between years 1974 and 2010, 920 men had died.

### Statistical analyses

The data are presented as means with SDs or as counts with percentages. Statistical significances for the unadjusted hypothesis of linearity across categories of AHA were evaluated using the Cochran–Armitage test for trend and analysis of variance with an appropriate contrast. In the case of violation of the assumptions (e.g. non-normality), a bootstrap-type test was used. To determine characteristics associated with AHA 6 or AHA 5–6, univariate and multivariate forward stepwise logistic regression analyses were applied. The normality of the variables was tested using the Shapiro–Wilk *W* test. Stata 15.1 (StataCorp LP, College Station, TX, USA) was used for the analyses.

## Results

The baseline characteristics of the cohort in 1974 are shown in Table 1. Almost the half of the study population (839/1759, 47.7%) was alive in 2010. Of the survivors in 2010, 10.0% (*n* = 84) fulfilled all AHA criteria (AHA 6), which is 4.7% (84/1759) of the study group. 13.7% of the survivors (*n* = 115) had chronic diseases but fulfilled other five criteria (AHA 5). The mean age of survivors with AHA 6 was 46 years at baseline, and mean age decreased linearly in AHA groups towards AHA 6. At baseline in 1974, of the men fulfilling all AHA criteria (AHA 6) 82% had been non-smokers, their mean BMI was 25.2 (kg/m^2)^, mean total cholesterol 6.0 (mmol/L), and mean consumption of alcohol 139 g/week. Furthermore, 69% of them reported their health as “very good” or “fairly good” at baseline in 1974.

**Table 1:** Age-adjusted characteristics of study population (*n* = 1759 ) in 1974

AHA in 2010 Variable in 1974	AHA 0 *n* = 920	AHA 1 *n* = 306	AHA 2–4 *n* = 334	AHA 5 *n* = 115	AHA 6 *n* = 84	*p* value for difference between AHA groups
Age, years, mean (SD)	49 (4)	47 (4)	47 (4)	46 (4)	46 (4)	< 0.001
BMI, kg/m^2^, mean (SD)	26.3 (2.9)	25.8 (2.7)	26.0 (2.8)	25.2 (2.4)	25.2 (2.7)	< 0.001
Non-smokers, *n* (%)	545 (59)	219 (72)	246 (74)	90 (78)	69 (82)	< 0.001
Blood pressure, mm Hg, mean (SD)						
Systolic	146 (20)	140 (17)	140 (18)	141 (20)	139 (16)	< 0.001
Diastolic	94 (12)	91 (11)	91 (11)	90 (12)	89 (10)	< 0.001
Serum lipids, mmol/L, mean (SD)						
Cholesterol	6.42 (1.05)	6.32 (1.03)	6.19 (1.06)	6.17 (0.98)	6.01 (1.05)	< 0.001
Triglycerides	1.77 (1.03)	1.59 (0.73)	1.69 (1.02)	1.46 (0.71)	1.50 (0.73)	< 0.001
Alcohol, g/week	178 (171)	158 (140)	150 (134)	126 (111)	139 (134)	< 0.001
Coffee, cups/day	4.2 (2.6)	3.7 (2.3)	4.3 (2.6)	3.9 (2.3)	3.7 (2.1)	0.082
SRH, *n* (%)^a^	438 (48)	173 (57)	178 (53)	80 (70)	58 (69)	< 0.001
SRF, *n* (%)^a^	287 (31)	125 (41)	104 (31)	43 (37)	38 (45)	0.020

Continuous variables are mean (SE)

*BMI* body mass index, *SRH* self-rated health, *SRF* self-rated fitness

^a^Assessed as excellent or very good

Overall, AHA number in 2010 was linearly associated (*p* < 0.001) with age, lower BMI, lower systolic and diastolic blood pressure, and lower levels of total cholesterol and triglycerides at baseline in midlife. In addition, AHA was associated with moderate consumption of alcohol (*p* < 0.001), higher SRH (*p* < 0.001) and self-rated fitness (*p* = 0.020), and inversely associated with smoking (*p* < 0.001) in midlife.

In logistic regression analysis adjusted for age, the midlife factors of preventing healthy aging without chronic diseases (AHA 6) were smoking (OR 0.44, 95% CI 0.25–0.77; *p* = 0.005), higher BMI (OR 0.75, 0.59–0.96; *p* = 0.021), and higher total cholesterol (OR 0.76, 0.60–0.97; *p* = 0.030). In addition, SRH (OR 1.73, 1.07–2.80; *p* = 0.026) assessed as “very good” or “fairly good” in midlife was statistically significantly associated with increased odds of fulfilling all AHA criteria (Table 2).

**Table 2:** Odds for active and healthy aging (fulfilling six AHA criteria) among the HBS cohort

Variable	Univariate		Multivariate^a^	
	OR (95% CI)	*p* value	OR (95% CI)	*p* value
Age	0.88 (0.83–0.93)	< 0.001	0.89 (0.84–0.94)	< 0.001
Smoking	0.42 (0.24–0.73)	0.002	0.44 (0.25–0.77)	0.005
Systolic blood pressure	0.74 (0.58–0.94)	0.015		
Diastolic blood pressure	0.77 (0.61–0.67)	0.025		
Body mass index	0.72 (0.57–0.91)	0.007	0.75 (0.59–0.96)	0.021
Total cholesterol	0.72 (0.57–0.90)	0.005	0.76 (0.60–0.97)	0.030
Triglycerides	0.76 (0.57–1.01)	0.061		
Alcohol	0.81 (0.62–1.06)	0.12		
Coffee	0.83 (0.65–1.05)	0.12		
Self-rated health (SRH)	2.07 (1.29–3.32)	0.003	1.73 (1.07–2.80)	0.026
Self-rated fitness (SRF)	1.65 (1.06–2.56)	0.026		

Full AHA criteria: (1) Being alive, (2) responding to the postal questionnaire survey, (3) no subjective cognitive problems, (4) feeling of happiness, (5) no difficulties in activities of daily living (ADL), and (6) no significant chronic diseases

^a^Forward stepwise selection. Only those variables are shown which entered the model

Furthermore, smoking (OR 0.52, 0.36–0.77; *p* < 0.001) and higher BMI (OR 0.73, 0.61–0.86; *p* < 0.001) and higher consumption of alcohol (OR 0.73, 0.60–0.90; *p* = 0.003) at midlife were significantly inversely associated with fulfilling five AHA criteria with chronic diseases. In addition, SRH reported as “very good” or “fairly good” in midlife was statistically significantly associated with increased odds of fulfilling five AHA criteria (OR 1.90, 1.37–2.63; *p* < 0.001; Table 3).

**Table 3:** Odds for fulfilling five AHA criteria among the HBS cohort

Variable	Univariate		Multivariate^a^	
	OR (95% CI)	*p* value	OR (95% CI)	*p* value
Age	0.88 (0.84–0.91)	< 0.001	0.87 (0.84–0.91)	< 0.001
Smoking	0.46 (0.32–0.66)	< 0.001	0.52 (0.36–0.77)	< 0.001
Systolic blood pressure	0.81 (0.69–0.95)	0.009		
Diastolic blood pressure	0.80 (0.69–0.93)	0.004		
Body mass index	0.70 (0.60–0.82)	< 0.001	0.73 (0.61–0.86)	< 0.001
Total cholesterol	0.78 (0.67–0.91)	0.002		
Triglycerides	0.71 (0.58–0.87)	< 0.001		
Alcohol	0.73 (0.60–0.88)	< 0.001	0.73 (0.60–0.90)	0.003
Coffee	0.87 (0.74–1.01)	0.076		
Self-rated health (SRH)	2.21 (1.61–3.04)	< 0.001	1.90 (1.37–2.63)	< 0.001
Self-rated fitness (SRF)	1.39 (1.02–1.88)	0.033		

Five AHA criteria: (1) Being alive, (2) responding to the postal questionnaire survey, (3) no subjective cognitive problems, (4) feeling of happiness, (5) no difficulties in activities of daily living (ADL), with chronic diseases

^a^Forward stepwise selection. Only those variables are shown which entered the model

## Discussion

Our findings showed that cardiovascular risk factors and SRH in midlife are associated with AHA in our businessmen cohort. Non-smoking, lower BMI and total cholesterol levels, and higher SRH in midlife predicted fulfilling all six AHA criteria, that is, healthy aging without chronic diseases. Whilst, non-smoking, lower BMI and alcohol consumption, and higher SRH in midlife were statistically significantly associated with fulfilling five AHA criteria, which include the possibility of one or more chronic diseases. AHA was also linearly associated with lower levels of systolic and diastolic blood pressure, lower triglycerides, and higher SRF in midlife, although these were not predictors of AHA in multivariate analysis.

Our results of AHA as a general concept are in accordance with studies on successful aging, which have shown the importance of various midlife behavioral and biomedical factors on longevity [12, 15, 24]. Non-smoking in midlife was a strong predictor of AHA in the HBS cohort. Similarly, smoking has been associated with late-life disability in several studies [9, 26–28]. Previous studies have also shown that obesity in midlife is associated with disability in old age [8, 9, 25–27]. A recent study suggested that participants with normal weight in midlife had more healthy years without diseases than obese participants [28]. Our results showed that lower BMI in midlife predicted the fulfillment of all six AHA criteria, but also AHA with chronic diseases, which implies the benefits of current medical care and treatments. Lower levels of total cholesterol in midlife predicted AHA without diseases in our study. This finding is consistent with previous studies, which have reported an association between midlife high cholesterol and cognitive decline or dementia in later life [29, 30]. High serum cholesterol and systolic blood pressure, and especially a combination of these, have been reported as midlife risk factors of Alzheimer disease in old age [31]. Sabia et al. [11], and Pruchno and Wilson-Genderson [27] have suggested that moderate consumption of alcohol is associated with successful aging. This is in line with our results, although, lower consumption of alcohol predicted five AHA criteria, with chronic diseases. In contrast to previous studies on longevity [9, 16, 19, 32], blood pressure was not an independent predictor of AHA in our analyses. However, there was a linear association between lower blood pressure in midlife and AHA, and the blood pressure variables were in the univariate analyses of predictivity, but they did not enter into the multivariate analyses. This can be explained by missing blood pressure data in 1974, and also by correlation of blood pressure with age, BMI and smoking. In addition, an antihypertensive treatment during follow-up may also have diluted the differences.

Furthermore, earlier studies of the HBS cohort have shown associations between cardiovascular risk factors in midlife and health-related quality of life in old age [16–19]. However, the cross-sectional study in old age [7] suggested that cardiovascular risk factors were not significantly related to AHA, which may be due to reverse causation and the effects of medications.

Our results on SRH as a predictor of AHA support observations of SRH as an important marker of health status and successful aging, but also an independent predictor of mortality [33, 34]. In addition, previous results from the HBS cohort indicated that poor SRH in clinically healthy midlife was related to frailty in old age [21].

To our knowledge, longitudinal studies of successful aging in spite of chronic diseases are scarce. Our study population consists of men who were certified to be clinically healthy in baseline, but morbidity increased during follow-up. Advanced treatment and cure of diseases may enable an individual to age “successfully” and maintain functionality in old age. However, recent studies have suggested that absence of disease is not the most important element of successful aging, and more emphasis should be focussed on psychological and social factors [35]. Some concern has been expressed that Rowe and Kahn’s model with binary interpretation might not be the most appropriate model for assessing successful aging in people with chronic illnesses [6, 36]. Young et al.’s [3] model of successful aging takes into account adaptive psychological and social mechanisms to compensate limitations of physiological health. Manierre [37] has tested the validity of Young et al.’s [3] model and demonstrated that it provides more holistic perspective of successful aging, especially among people with chronic diseases. The criteria of AHA used in the HBS cohort [7] capture the psychological, physical, and social components of successful aging as defined by Rowe and Kahn [2]. However, the AHA criteria include also the question of happiness which could describe a dimension of emotional vitality as proposed by Young et al. [3]. Our study showed that fulfilling the criteria of psychological and social dimensions were not dependent on the absence of diseases.

The concept of healthy aging has induced much debate as longevity has increased, and various definitions of successful aging have been introduced [6, 24, 38]. The life expectancy for men from same birth cohort as our study group was only 50.2 years (mean), while currently the life expectancy for men in Finland is 78.7 years [39]. Survivors of our cohort were at mean age of 82 in 2010 which is already beyond average life expectancy, even among the highest socioeconomic group [40]. The important aspect is how to expand healthy and active years in old age. According to Kleinedam et al. [38], well-constructed operationalization of successful aging includes measurements of physiological health, well-being and social engagement, with subjective and objective aspects. In the present study, the criteria of AHA [7] were based on self-reports in mailed questionnaires, which included both objective and subjective indicators. In the AHA criteria, responding to the mailed questionnaire survey reflected social functioning and contribution to the society.

The previous cross-sectional study of the HBS cohort showed that about 5% of men met all criteria for AHA, which may infer the strictness of assessment [7]. In our longitudinal study, 4.7% of the study population and 10% of the survivors in 2010 fulfilled all AHA criteria and can be defined as active and healthy agers. The proportion of successful agers have ranged widely across the studies; in the review of Depp and Jeste [24], the mean proportion was 36%. Anyway, active and healthy aging is not a common phenomenon. Our results, with strict definition of AHA [7], indicated the importance of midlife factors on longevity and functional aging.

The strength of our study is the very long, over 40-year follow-up of initially healthy men. Our prospective study has also taken into account the effect of mortality in the analyses of AHA, whereas earlier studies have provided only analyses of survivors. Our study sample is very homogeneous representing men from the upper social class which reduces confounding by socioeconomic factors. On the other hand, a socioeconomically homogeneous population of white men is a limitation for generalizing the results. Moreover, future research could examine in more detail the association of personal characteristics, such as resilience, with AHA.

Our results suggest that having a healthy lifestyle in midlife, especially avoiding smoking and obesity, may improve the probability of AHA and, therefore, the results give new and important incentives for promoting such lifestyle. Understanding of the long-term aspects and midlife predictors of AHA can be used for health policies and for increasing successful aging in populations of aging societies.
